# Clonal Mosaicism of Mitochondrial DNA Heteroplasmy as a Molecular Clock of Aging

**DOI:** 10.1111/acel.70718

**Published:** 2026-09-11

**Authors:** Renin Chang, Andy P. Tsai, Boyang Wang, Kuan‐Hao Tsui, Cheng‐Yoong Pang, Chia‐Jung Li

**Affiliations:** ^1^ Department of Medical Education and Research Kaohsiung Veterans General Hospital Kaohsiung Taiwan; ^2^ Center of General Education Cheng Shiu University Kaohsiung Taiwan; ^3^ Center of General Education Shu‐Zen Junior College of Medicine and Management Kaohsiung Taiwan; ^4^ Department of Neurology and Neurological Sciences Stanford University School of Medicine Stanford California USA; ^5^ Sundial Initiative Palo Alto California USA; ^6^ Department of Obstetrics and Gynecology Kaohsiung Veterans General Hospital Kaohsiung Taiwan; ^7^ Institute of Biopharmaceutical Sciences National Sun Yat‐Sen University Kaohsiung Taiwan; ^8^ Department of Medical Research Hualien Tzu Chi Hospital, Buddhist Tzu Chi Medical Foundation Hualien Taiwan; ^9^ Center for Cell Therapy and Development Hualien Tzu Chi Hospital Hualien Taiwan; ^10^ National Museum of Marine Biology & Aquarium Pingtung Taiwan

**Keywords:** aging, clonal expansion, heteroplasmy, mitochondrial DNA, molecular clock, respiratory chain deficiency, somatic mutation

## Abstract

The accumulation of somatic mitochondrial DNA (mtDNA) mutations across life is among the oldest and most debated proposed drivers of aging. A defining, counter‐intuitive feature is that individual mutant molecules, although vanishingly rare when they arise, can come to dominate a cell's multi‐copy mtDNA population through intracellular clonal expansion, producing a mosaic of respiratory‐deficient cells across aging tissues. Here we synthesize current evidence to argue that clonal mosaicism of mtDNA heteroplasmy constitutes a quantifiable, tissue‐specific molecular clock of aging. We trace foundational single‐cell and multi‐tissue observations of somatic mtDNA mutation, examine the causal evidence from mtDNA mutator mice, and dissect the debate between neutral genetic drift and cellular selection that governs clonal expansion. We then integrate recent single‐cell and population‐scale studies that have transformed the field: deep multi‐tissue surveys revealing tissue‐specific accumulation and a biphasic signature, biobank analyses linking heteroplasmy burden to mortality and organ‐specific disease, and a two‐step mechanism in which cryptic replication‐error mutations become detectable through age‐related clonal mosaicism. We discuss technologies such as single‐cell mtDNA genotyping, duplex and long‐read sequencing, and droplet digital PCR that now read the clock at single‐molecule resolution, and we connect mutational accumulation to downstream aging phenotypes through mtDNA‐driven innate immune signaling, cellular senescence and inflammaging. Finally, we position the mitochondrial clock alongside epigenetic and other aging clocks, highlighting concordance, complementarity, and what must be resolved before heteroplasmy can serve as a blood‐based biomarker of biological age.

## Graphical abstract

Mitochondrial DNA mutations diverge by tissue dynamics across lifespan: dividing tissues accumulate pervasive point mutations via clonal stem‐cell expansion, whereas post‐mitotic tissues harbor clonally expanded large‐scale deletions, both culminating in age‐dependent, threshold‐limited respiratory failure.

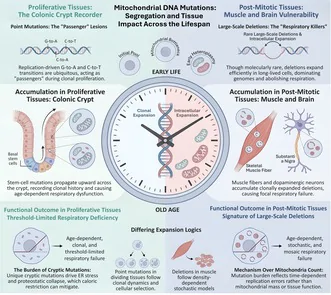

## 1 Introduction

Biological aging is increasingly understood as the time‐dependent accumulation of molecular damage across a set of interconnected cellular pathways, of which mitochondrial dysfunction is consistently listed among the central hallmarks (Lopez‐Otin et al. 2023). Within this framework, the mitochondrial genome occupies a peculiar and informative position. Unlike the diploid nuclear genome, mtDNA is present at hundreds to thousands of copies per cell, is maternally inherited, and can exist in a mixed state, heteroplasmy, in which mutant and wild‐type molecules coexist within the same cell, tissue, and individual (Stewart and Chinnery 2021). This high ploidy means that a newly arising somatic mutation is initially diluted to near‐undetectable levels and produces no biochemical consequence because the surrounding wild‐type genomes complement its defect (Stewart and Chinnery 2021). Indeed, accumulation of mtDNA mutations is now regarded as one of the strongest molecular signatures of aging (Gupta et al. 2026).

The central puzzle, and the reason mtDNA heteroplasmy is so useful as a record of time, is that such rare mutant molecules do not remain rare. In many aging cells, they undergo clonal expansion, progressively displacing the wild‐type population until they predominate and impose a respiratory‐chain defect (Stewart and Chinnery 2021). The result is a mosaic tissue in which scattered single cells are respiratory‐deficient while their neighbors are unaffected. A defining recent insight is that the bulk of these events are “cryptic”, unique to single cells and invisible to aggregate assays, yet accumulating in a manner that coincides with mid‐to‐late life and with species lifespan (Green et al. 2025). This pattern, cell‐by‐cell, accumulating with chronological age, written into a faithfully copied and segregated genome, has the essential properties of a molecular clock.

In this review we develop the argument that clonal mosaicism of mtDNA heteroplasmy can be read as a quantitative, tissue‐resolved clock of aging. The proposition has acquired new force from single‐cell and population‐scale studies, culminating in the demonstration that mtDNA clonal mosaicism encodes a biphasic, tissue‐specific signature of chronological age (Wang, Li, et al. 2025), and in the resolution of a two‐step mechanism by which cryptic replication‐error mutations become detectable through age‐related clonal mosaicism in human blood (Gupta et al. 2026). We first establish the foundational evidence, then turn to causality, mechanism, tissue specificity, population dynamics, enabling technologies, and the link to aging phenotypes, before comparing the mitochondrial clock with other emerging clocks of biological age.

## 2 The Mitochondrial Genome: Copy Number, Heteroplasmy and the Threshold Effect

The ~16.5‐kb human mtDNA encodes 13 polypeptides of the oxidative phosphorylation machinery, together with the rRNAs and tRNAs required for their intra‐mitochondrial synthesis (Stewart and Chinnery 2021). Three features make this genome unusually prone to becoming a mosaic record of somatic events. First, its copy number is high and variable between cell types, so that any individual mutation begins as a tiny fraction of the cellular pool; population‐scale sequencing confirms that nearly every human carries heteroplasmic variants, that heteroplasmic single‐nucleotide variants tend to arise somatically and accumulate sharply after about the age of 70, and that copy number declines with age and is under partial nuclear genetic control (Gupta et al. 2023). Second, mtDNA is replicated continuously, even in post‐mitotic cells, and its dominant age‐accumulating mutational signature (G to A/C to T transitions) is indicative of replication errors rather than oxidative damage (Sanchez‐Contreras et al. 2023). Third, because the molecule is multi‐copy, the genotype–phenotype relationship is non‐linear: functional complementation buffers low‐level heteroplasmy, and a biochemical defect appears only once a mutant clone crosses the threshold for that mutation (Stewart and Chinnery 2021).

This threshold behavior explains an apparent paradox that has long shaped the field: average heteroplasmy levels measured in bulk tissue are often very low, seemingly too low to matter, even though individual cells can be near‐homoplasmic for a deleterious variant and frankly respiratory‐deficient (Hinton Jr. et al. 2024). The resolution is that the biologically relevant quantity is not the tissue mean but the per‐cell distribution, and in particular the fraction of cells that have crossed threshold. It is precisely this mosaicism, rather than a uniform decline in mitochondrial mass, that distinguishes mtDNA‐driven aging and that makes the underlying genetics legible as a per‐cell clock.

## 3 Somatic mtDNA Mutations Accumulate and Clonally Expand with Age

### 3.1 Tissue‐Specific Accumulation Across the Body

**FIGURE 1:**
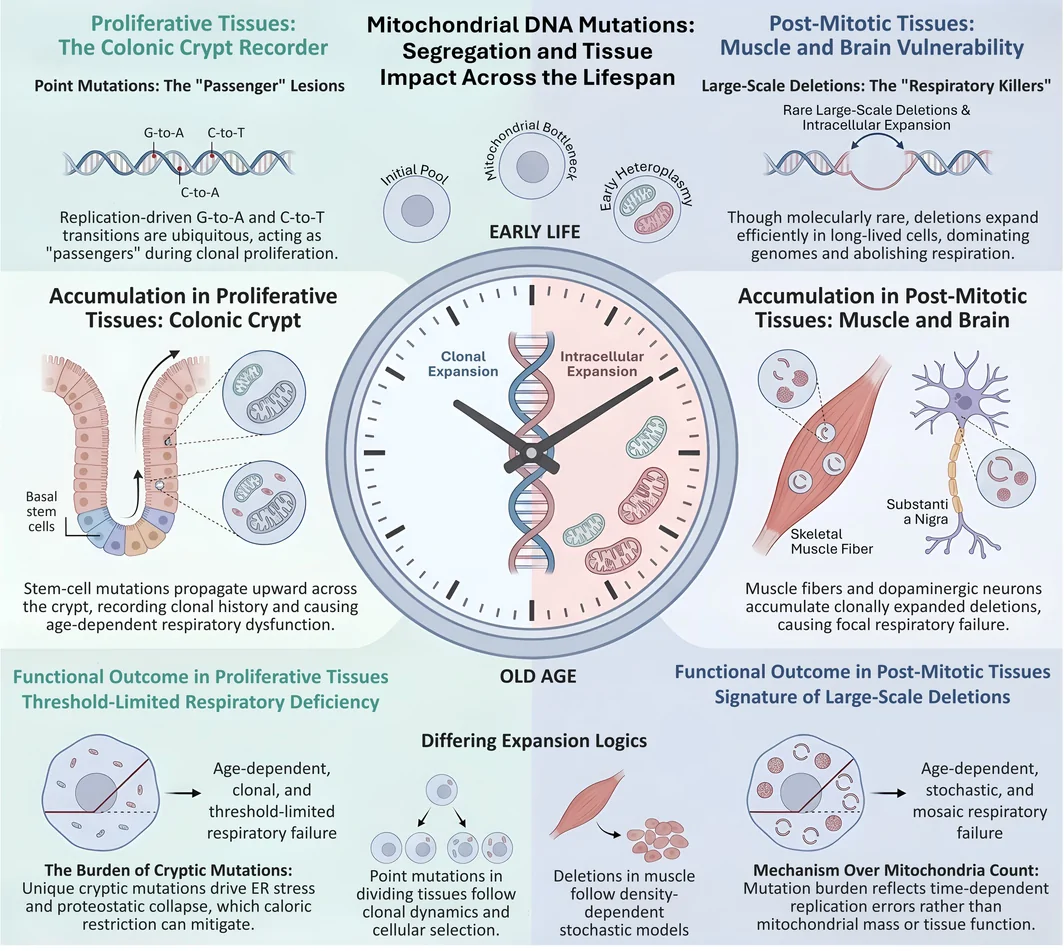
Clonal mosaicism of mtDNA heteroplasmy as a biphasic, tissue‐specific clock of aging. In proliferative tissues (colonic crypt), replication‐error point mutations act as passengers, become fixed in stem cells, and produce threshold‐limited respiratory deficiency; in post‐mitotic tissues (muscle, substantia nigra), rare large‐scale deletions expand within single cells to abolish respiration. The two arms follow different expansion logics but converge on a mosaic of respiratory‐deficient cells.

High‐accuracy sequencing is only beginning to map somatic mtDNA mutation across the body (Figure 1), and the most comprehensive dataset to date remains murine: duplex sequencing of eight tissues from an aging mouse cohort cataloged more than 89,000 independent somatic mtDNA mutations, revealing significant tissue‐specific increases with age that did not track mitochondrial content or tissue function, and confirming that the age‐accumulating signature reflects replication errors rather than reactive oxygen species (Sanchez‐Contreras et al. 2023). The corresponding human picture relies primarily on single‐cell (droplet‐based) sequencing studies, which provide useful but still incomplete mutation profiling restricted to a handful of tissues, so cross‐tissue human coverage lags substantially behind the mouse data. In parallel, single‐cell analyses across human and rodent tissues established that the dominant fraction of the somatic burden consists of cryptic mutations unique to individual cells, which are mechanistically linked to endoplasmic‐reticulum stress, disrupted proteostasis and immune/inflammatory gene expression, and which can be slowed by caloric restriction (Green et al. 2025). Direct sequencing of normal human somatic cells has likewise shown that mtDNA mosaicism is pervasive in healthy tissue and accrues with age (An et al. 2024).

Post‐mitotic, long‐lived cells provide clear single cell demonstrations of these processes. In skeletal muscle, single fibers accumulate clonally expanded mtDNA deletions. The expansion mechanism of these deletions has been debated for three decades and can be explained without invoking a replicative advantage (Insalata et al. 2022). These deletions can also be quantified fiber by fiber using droplet digital PCR to link clonal deletion load directly to focal respiratory failure (Trifunov et al. 2018). In the brain, dopaminergic neurons of the substantia nigra were shown to accumulate high levels of clonally expanded mtDNA deletions during normal aging, with deletion burden significantly higher in respiratory‐deficient than respiratory‐normal neurons, implicating the expanded mutation directly in the defect (Bender et al. 2006); contemporary reviews place this neuronal mtDNA instability within the broader landscape of central‐nervous‐system aging and neurodegeneration (Steffan et al. 2025).

### 3.2 Proliferative Tissues: The Colonic Crypt as a Clonal Recorder

Replicating tissues convert mtDNA mutation into a visible clonal history. The human colonic crypt is maintained by a few basal stem cells whose progeny migrate upward, so a mutation fixed in a stem cell is propagated through the entire crypt. Comprehensive analysis of aging colorectal epithelium showed that clonally expanded point mutations arise from early to mid‐life and drive a substantial burden of respiratory‐chain dysfunction by old age (Greaves et al. 2014). Because adjacent respiratory‐deficient cells are clonally derived, naturally occurring mtDNA mutations act as endogenous lineage labels, and their distribution has been used to quantify human intestinal stem‐cell number and crypt fission dynamics in normal and neoplastic colon (Baker et al. 2019). The convergence of proliferative and post‐mitotic tissues on the same outcome, age‐dependent, clonal, threshold‐limited respiratory deficiency, is the empirical core of the mitochondrial clock concept.

### 3.3 Two Classes of Somatic Lesion: Point Mutations and Deletions

Two mechanistically distinct classes of somatic lesion contribute to the clock, and they behave differently. Point mutations, dominated by the G to A/C to T transitions characteristic of replication error, accumulate across essentially all tissues and make up the bulk of the cryptic single‐cell burden (Green et al. 2025; Gupta et al. 2026). They arise continuously, are carried as passengers when a clone proliferates, and become detectable in bulk tissue only once that clone has expanded (Gupta et al. 2026). Large‐scale deletions, by contrast, are comparatively rare at the molecular level but expand with particular efficiency in long‐lived post‐mitotic cells such as skeletal muscle fibers and substantia nigra neurons, where a single deleted species can come to occupy most of the genomes in a cell and abolish respiration (Bender et al. 2006; Zhang et al. 2018). The two classes also differ in their expansion logic: deletion expansion in muscle is well described by density‐dependent stochastic models that require no replicative advantage (Insalata et al. 2022), whereas point‐mutation accumulation in proliferative tissue is governed by clonal dynamics and, increasingly, by demonstrable cell‐level selection (Kotrys et al. 2024). A faithful clock must therefore track both classes separately, because their tissue distributions, rates and functional thresholds are not interchangeable; conflating them is one source of the long‐standing discrepancy between low average heteroplasmy and frank single‐cell respiratory failure (Hinton Jr. et al. 2024).

It is important to emphasize that this tissue partition is a spectrum rather than an absolute dichotomy, and that the apparent segregation of lesion classes by proliferative status partly reflects a detection bias: unlike point mutations, deletions are structurally heterogeneous and are not well captured by duplex‐sequencing panels or single‐cell genotyping schemes built around defined single‐nucleotide positions, so their burden outside the classic post‐mitotic tissues is comparatively under‐ascertained. Where deletions have been specifically sought in proliferative tissue, they are readily found: UV‐exposed human epidermis and dermis accumulate large‐scale mtDNA deletions, including the well‐characterized “common deletion,” in proportion to cumulative sun exposure (Birch‐Machin et al. 1998; Eshaghian et al. 2006; Pang et al. 1994), and deletions are also detectable, albeit less frequently than point mutations, in aging colonic epithelium (An et al. 2024). Notably, deletion burden in the proliferative epidermis appears constrained relative to the less proliferative dermis, consistent with selection against deletions under sustained cell turnover (Pang et al. 1994). Conversely, point mutations are not restricted to proliferative tissue: they accumulate, generally at lower burden than in blood or gut, in post‐mitotic skeletal muscle and other high‐energy tissues as part of the same replication‐error signature described above (Sanchez‐Contreras et al. 2023). The clock is therefore better understood as two lesion classes whose relative dominance shifts gradually with a tissue's proliferative status and turnover‐dependent selective pressure, rather than as two lesion classes confined to mutually exclusive tissue compartments.

## 4 Mechanisms and Dynamics of the Mitochondrial Clock

### 4.1 Causality: Lessons From the mtDNA Mutator Mouse

**FIGURE 2:**
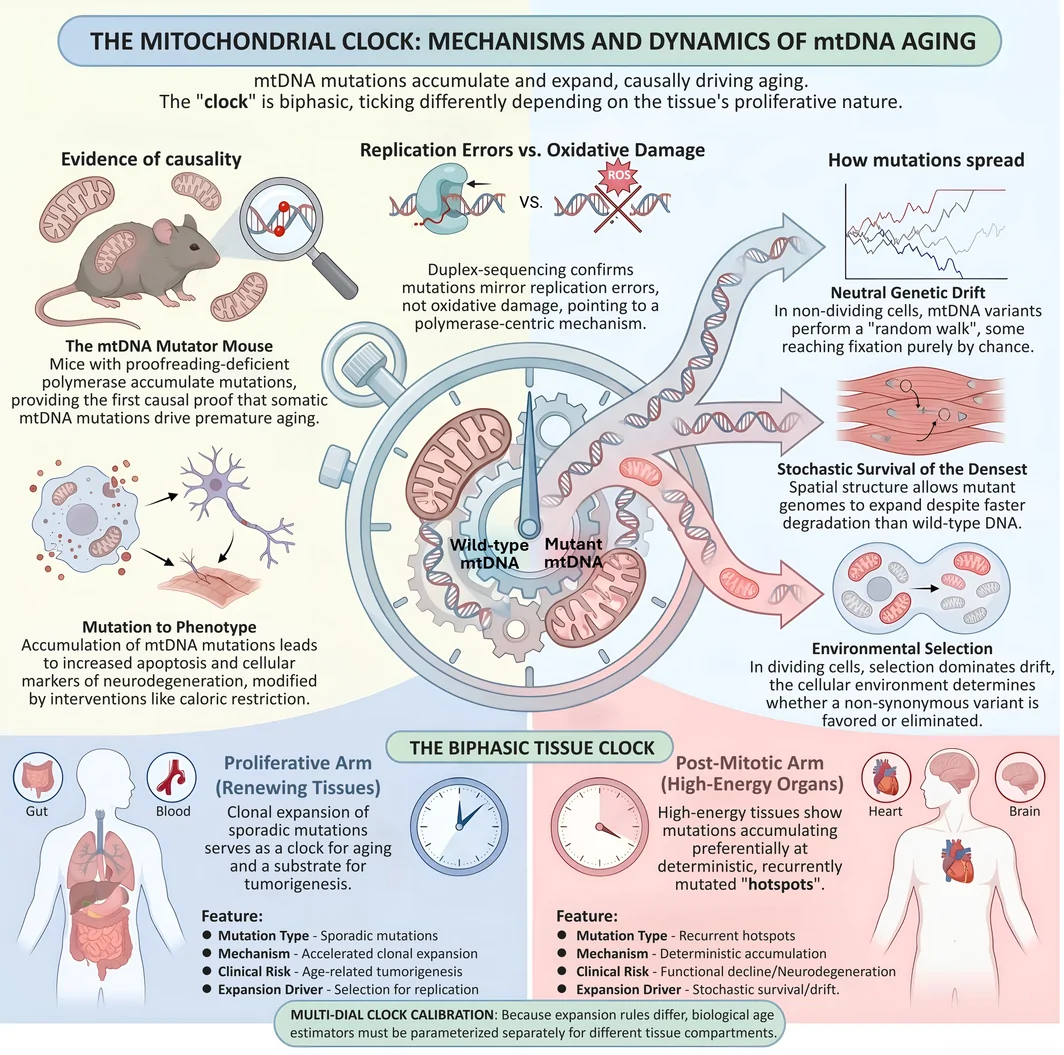
Mechanisms and dynamics of the mitochondrial clock. Causal evidence from the mtDNA mutator mouse and a replication‐error (not oxidative) mutational signature point to a polymerase‐centric mechanism, while clones spread by neutral drift, stochastic survival of the densest, or environment‐dependent selection. The resulting clock is biphasic, with a proliferative arm (tumorigenesis risk) and a post‐mitotic, hotspot‐driven arm (functional decline), requiring tissue‐specific calibration.

Whether somatic mtDNA mutations cause, rather than merely accompany, aging was contested for decades. The decisive experimental test came from mtDNA “mutator” mice expressing a proofreading‐deficient mitochondrial polymerase. Homozygous mutator mice accumulate a markedly increased load of point mutations and deletions and develop a premature‐aging phenotype, providing the first causal evidence that somatic mtDNA mutations are sufficient to drive mammalian aging phenotypes (Trifunovic et al. 2004); an independent mutator line linked mutation accumulation to apoptosis in aging tissues (Kujoth et al. 2005). Complementing these homozygous models, mice heterozygous for the same proofreading‐deficient polymerase allele (PolgA+/mut) do not develop overt premature aging but nonetheless accumulate clonally expanded mtDNA point mutations with age in colonic crypts, largely without the gross structural rearrangements or copy‐number instability that characterize the homozygous mutator, and reproduce a respiratory‐deficiency phenotype that closely parallels aged human colon (Baines et al. 2014). This dose‐graded model helps isolate the contribution of point‐mutation clonal expansion from that of broader structural genomic instability. These remain the foundational causal experiments in the field, and recent work has extended their logic. A multi‐tissue duplex‐sequencing survey confirmed that the mutational signatures accumulating with natural aging mirror replication‐error processes rather than oxidative damage, consistent with the polymerase‐centric mechanism (Sanchez‐Contreras et al. 2023), and single‐cell studies across species have shown that cryptic mutation burden correlates with markers of aging and neurodegeneration and is modifiable by interventions such as caloric restriction (Green et al. 2025; Figure 2).

### 4.2 Mechanisms of Clonal Expansion: Drift Versus Selection

How a single mutant molecule comes to dominate a cell containing thousands of genomes determines how the clock should be calibrated, and the relative contributions of neutral drift and selection remain actively debated (Schaack et al. 2020). Because mtDNA is continually turned over and randomly partitioned, the frequency of any variant performs a random walk, so some variants drift toward fixation by chance even in non‐dividing cells. A refinement of the neutral framework, stochastic survival of the densest, shows that mutant genomes can rise in mean frequency in spatially structured systems even when degraded slightly faster than wild type, accounting for deletion expansion in muscle without an explicit replicative advantage (Insalata et al. 2022).

Recent single‐cell work has sharpened the role of selection. Combining precise mtDNA base editing with ultra‐high‐throughput single‐cell heteroplasmy tracking showed that in dividing cells selection, rather than simple drift, dominates the shaping of population heteroplasmy, with the sign of selection on a non‐synonymous variant being wholly dependent on the cellular environment (Kotrys et al. 2024). Single‐cell multi‐omic profiling of human immune cells carrying pathogenic variants revealed dynamic purifying selection that varies across cell types (Lareau et al. 2023), and high‐throughput single‐cell genotyping has uncovered functional, high‐frequency mtDNA mutations that were invisible to bulk methods (Guo et al. 2023). Computational and single‐cell dissection further indicates that many subpopulation‐specific variants are pre‐existing heteroplasmies carried as passengers during clonal expansion rather than de novo somatic events (Wang, Wang, et al. 2025). The contemporary synthesis is that drift and selection are not mutually exclusive but operate in different tissues and contexts. Functionally, induced pluripotent stem‐cell models engineered to high deleterious heteroplasmy show altered growth, metabolic shifts and increased epigenetic age in their differentiated progeny—a cell‐intrinsic link demonstrated to date only in this stem‐cell system, rather than a general finding across somatic tissues (Vandiver et al. 2025).

### 4.3 A Biphasic, Tissue‐Specific Clock

If clonal expansion follows different rules in different tissues, the clock it produces should be tissue‐specific, and recent data confirm this. Profiling of mitochondrial nucleic acids across dozens of human tissues from hundreds of individuals revealed that mtDNA clonal mosaicism develops with two distinct, tissue‐dependent aging signatures, supporting a biphasic model of the mitochondrial clock (Wang, Li, et al. 2025). The tissue‐specific patterns of lesion type, expansion mechanism, and age‐related readout across the body are summarized in Table 1. In proliferative tissues with constant turnover, aging is marked by accelerated accumulation of sporadic mutations and their clonal expansion, with implications for age‐related tumorigenesis; in post‐mitotic, high‐energy tissues such as heart and brain, mutations accumulate preferentially at deterministic, recurrently mutated hotspots (Wang, Li, et al. 2025). The multi‐tissue mouse survey similarly found tissue‐specific rates of accumulation and removal that did not simply mirror mitochondrial content (Sanchez‐Contreras et al. 2023). This biphasic architecture rationalizes previously disparate observations. The stochastic, drift‐influenced expansion seen in proliferative epithelium and the hotspot‐biased accumulation seen in neurons and cardiomyocytes are not contradictory but the two coordinated arms of one biphasic, tissue‐resolved clock. It also constrains clock calibration: a heteroplasmy‐based estimator of biological age must be parameterised separately for proliferative and post‐mitotic compartments, and the same chronological age will be written differently into blood, gut, muscle and brain. Recognizing the mitochondrial clock as intrinsically multi‐dial, rather than a single universal counter, and treating heteroplasmy as structured information rather than noise, is the central conceptual advance underpinning this review (Hinton Jr. et al. 2024).

**TABLE 1:** Tissue‐specific patterns of somatic mtDNA mutation accumulation and clonal expansion in aging: The two arms of the mitochondrial clock.

Tissue	Clock arm	Predominant lesion	Hallmark age‐related readout	References
Colonic/intestinal crypt epithelium	Proliferative	Point mutations (stem‐cell origin)	COX‐deficient crypts rising with age; reduced proliferation and increased apoptosis	Baker et al. (2019), Greaves et al. (2014)
Skin and other renewing epithelia	Proliferative	Point mutations (predominant); deletions also occur, counter‐selected	Accelerated accumulation and tumorigenesis risk; deletions rise with sun exposure but remain suppressed	Birch‐Machin et al. (1998), Eshaghian et al. (2006), Pang et al. (1994), Sanchez‐Contreras et al. (2023), Wang, Li, et al. (2025)
Skeletal muscle fibers	Post‐mitotic (segmental)	Large‐scale deletions	Mosaic COX‐deficient fibers; segmental respiratory failure; sarcopenia	Insalata et al. (2022), Trifunov et al. (2018)
Substantia nigra and other neurons	Post‐mitotic	Deletions (hotspot‐biased)	High deletion load; respiratory deficiency; links to neurodegeneration	Bender et al. (2006), Steffan et al. (2025)
Cardiomyocytes	Post‐mitotic	Recurrent hotspot mutations/deletions	Mosaic respiratory‐deficient cardiomyocytes; cardiac dysfunction	Wang, Li, et al. (2025)
Blood/hematopoietic system	Proliferative (clonal)	Heteroplasmic SNVs (cryptic, replication‐error)	Sharp post‐70 accumulation; associations with mortality, cancer and kidney disease	Gupta et al. (2026), Gupta et al. (2023), Hong et al. (2023), Huang et al. (2025)

Abbreviations: COX, cytochrome c oxidase; SNV, single‐nucleotide variant.

To avoid ambiguity, we use “one biphasic clock” to denote a unified conceptual model in which both arms arise from the same underlying process of intracellular clonal expansion, rather than referring to a single quantitative composite score. Point mutations and deletions differ in mutation rate, detectability, and expansion dynamics (Section 3.3), so a practical biological‐age estimator would need to combine lesion‐ and tissue‐specific inputs rather than pool them into one universal metric: for example, a point‐mutation‐burden term appropriate to blood and proliferative epithelia, and a deletion‐burden term appropriate to post‐mitotic muscle and brain, each fitted with its own rate parameters and combined only at the level of an interpretable summary score. Whether such integration is best achieved by simple weighted combination, by treating each tissue as an independent readout, or by some other scheme, and whether integration adds predictive value over separate tissue‐specific readouts is not yet established. We flag this explicitly as an open calibration problem rather than a solved feature of the framework.

The proliferative arm carries a distinctive risk signature. In constantly renewing epithelia the very clonal expansion that writes the clock also supplies raw material for neoplasia, and pan‐cancer data show that mtDNA truncating mutations can be positively selected in epithelial cancers of the kidney, colon, and thyroid (Yuan et al. 2020). The replication‐error mutational signature that dominates normal aging is likewise the signature seen in these tumors (Sanchez‐Contreras et al. 2023), suggesting that the proliferative clock and early tumorigenesis are not separate processes but two readouts of one underlying mutational engine (Wang, Li, et al. 2025). This dual character, at once a chronometer of aging and a substrate for cancer, is a defining and clinically important feature of the proliferative arm.

## 5 Population Dynamics, Mortality and the Inherited Baseline

### 5.1 Population‐Scale Heteroplasmy and Clinical Outcomes

**FIGURE 3:**
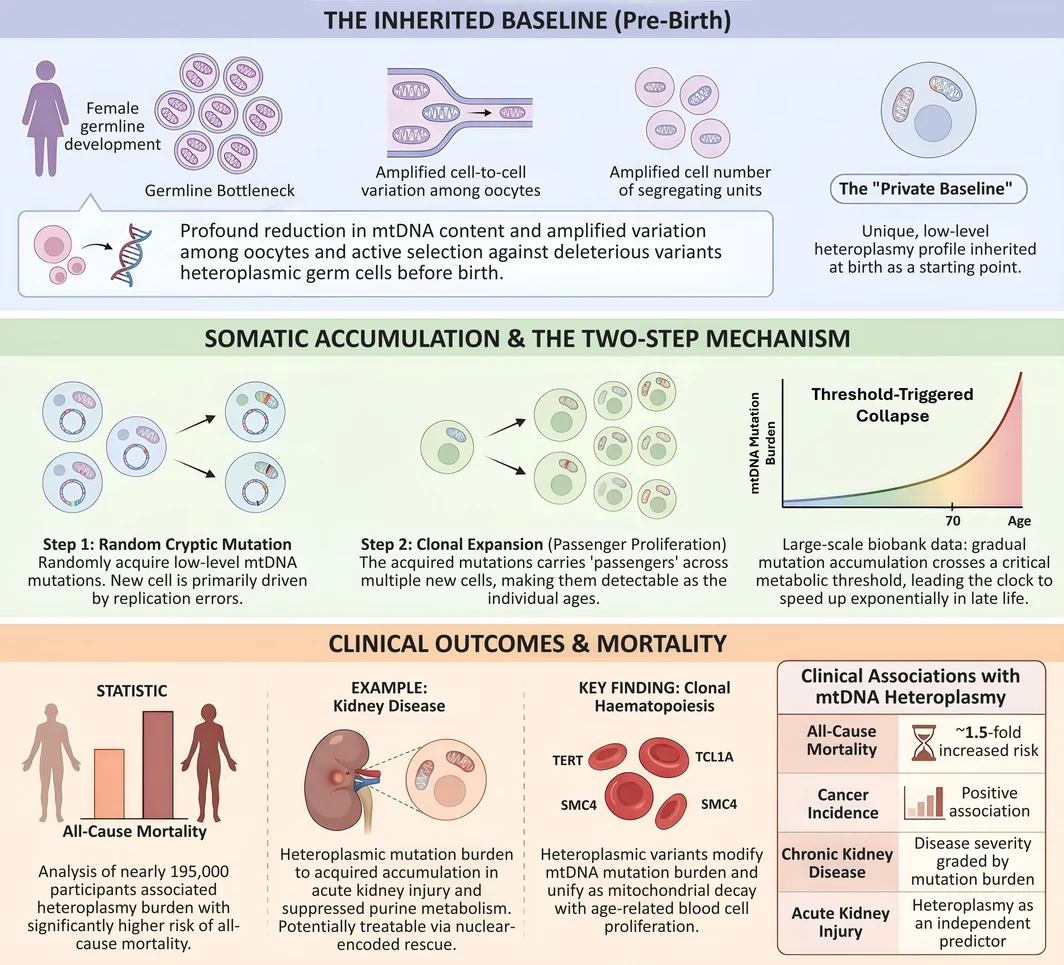
Inherited baseline, somatic accumulation, and clinical consequences of mtDNA heteroplasmy. A germline bottleneck sets a unique low‐level “private baseline” at birth. Somatic burden then accrues via a two‐step process: Random cryptic mutation followed by clonal expansion. This process crosses a threshold and accelerates after ~70 and is associated with all‐cause mortality, cancer, kidney disease, and clonal hematopoiesis (TERT, TCL1A, SMC4).

Biobank‐scale sequencing has moved heteroplasmy from a tissue‐biology curiosity to a population trait with clinical associations (Figure 3). Quantification of copy number and heteroplasmy across roughly 300,000 individuals showed sharp post‐70 accumulation of somatic heteroplasmic variants and identified nuclear loci controlling these traits, indicating that the rate of the clock is itself heritable and potentially modifiable (Gupta et al. 2023). The clinical relevance is direct: in nearly 195,000 UK Biobank participants, heteroplasmy and a constraint‐based functional score were associated with an approximately 1.5‐fold increased risk of all‐cause mortality and with cancer incidence and cancer‐specific mortality (Hong et al. 2023). A 2025 study went further, showing in 369,912 participants that heteroplasmic mutation burden grades with chronic kidney disease severity and independently predicts acute kidney injury, while mechanistically linking accumulated mutations to suppressed purine metabolism that is amenable to nuclear‐encoded rescue (Huang et al. 2025). Pervasive somatic mtDNA mosaicism in normal tissue may provide the cellular substrate for these population‐level associations; however, An et al., characterized clonal patterns in only a few cell types, so this link remains suggestive rather than definitive (An et al. 2024).

### 5.2 Longitudinal Change and the Two‐Step Mechanism

A clock must advance within an individual over time, not only across a cross‐section of ages. Prospective analyses now show that the variant allele fraction of deleterious heteroplasmies increases over time within individuals, rather than reflecting static drift, providing the temporal signature the clock interpretation requires (Kuiper et al. 2025). The mechanism behind this rise was clarified by genome‐wide analysis of mtDNA in the blood of roughly 750,000 individuals, which proposed a two‐step model: individual cells first randomly acquire low‐level cryptic mtDNA mutations, and these are then carried as passenger mutations when a cell clone proliferates, becoming detectable in whole blood with age. Germline variants near TERT, TCL1A, and SMC4, all linked to clonal hematopoiesis, modify this burden, and the mutational signature again points to replication errors rather than oxidative damage (Gupta et al. 2026, 2025). This unifies somatic mtDNA accumulation with clonal hematopoiesis and explains why the clock appears to accelerate in late life.

### 5.3 The Inherited Baseline and the Germline Bottleneck

Somatic accumulation begins from a baseline set before birth. During female germline development mtDNA passes through a genetic bottleneck in which a reduced effective number of segregating units amplifies cell‐to‐cell heteroplasmy variation among oocytes, a process whose somatic and developmental consequences have been reviewed in depth (van den Ameele et al. 2020; Zhang et al. 2018). Single‐cell deep sequencing of human primordial germ cells resolved this developmental bottleneck directly, showing a profound reduction in mtDNA content and a signature of selection against deleterious variants (Floros et al. 2018). Because bottleneck size and apparent selection are sensitive to sampling and statistical assumptions, careful estimators are required to avoid misleading inferences (Giannakis et al. 2023). The practical consequences for the aging clock are twofold: each individual inherits a private baseline of low‐level heteroplasmy upon which somatic events are superimposed, and the drift‐and‐selection forces that shape transmission also operate, in altered form, in the soma, so that germline and somatic heteroplasmy dynamics are mechanistically continuous.

## 6 Reading the Clock: Enabling Technologies

The interpretive power of the mitochondrial clock is bounded by the sensitivity and resolution of heteroplasmy measurement. Conventional short‐read sequencing is limited by intrinsic error and by nuclear mitochondrial pseudogenes, obscuring the low‐frequency variants that constitute the earliest reads of the clock. The principal approaches, together with their capabilities and limitations, are summarized in Table 2. Duplex sequencing suppresses errors to reveal rare somatic variants and their replication‐linked signatures across tissues (Sanchez‐Contreras et al. 2023), and droplet digital PCR provides absolute single‐cell or single‐fiber quantification of defined variants (Trifunov et al. 2018), and long‐read individual‐molecule sequencing preserves haplotype information that fragment‐based methods miss; the iMiGseq approach validated this in single cells but relies on PCR amplification of the mtDNA template (Bi et al. 2023), whereas subsequently developed Cas9‐guided nanopore methods sequence native, amplification‐free full‐length mtDNA molecules and further reduce amplification bias (Keraite et al. 2022; Vandiver et al. 2022). The most transformative development has been single‐cell mtDNA genotyping. The discovery that somatic mtDNA mutations behave as natural genetic barcodes, detectable in single‐cell sequencing data, enabled lineage tracing in humans with simultaneous readout of cell state (Ludwig et al. 2019). Massively parallel platforms coupling mitochondrial genotyping to chromatin accessibility, and enrichment methods that recover informative variants from droplet single‐cell RNA‐sequencing, have made single‐cell heteroplasmy measurement routine (Lareau et al. 2021; Miller et al. 2022), while multi‐omic single‐cell profiling resolves the selection dynamics acting on variants across cell types (Lareau et al. 2023). Applied longitudinally, these tools track clonal mosaicism in human hematopoiesis over time, directly observing the clonal expansion the clock model predicts (Lareau et al. 2019). Because the dominant somatic burden is cryptic and cell‐unique, single‐cell resolution is not a luxury but a prerequisite: aggregate assays average away precisely the signal that carries the clock (Green et al. 2025).

**TABLE 2:** Methods for detecting and quantifying mtDNA heteroplasmy and clonal mosaicism.

Method	Principle	Key capability	Main limitation	References
Bulk short‐read NGS	Massively parallel sequencing of pooled mtDNA	Genome‐wide heteroplasmy at population/biobank scale	Error floor ~0.1%–1%; NUMT contamination; averages away the per‐cell signal	Gupta et al. (2023)
Duplex sequencing	Tags and compares both DNA strands to suppress errors	Detection of ultra‐rare variants and true low‐frequency burden and signatures	High cost and depth requirement; no single‐cell context	Sanchez‐Contreras et al. (2023)
ddPCR	Absolute, partitioned quantification of a defined variant	Precise single‐fiber/single‐cell quantification of known deletions or mutations	Targeted (one variant per assay); not a discovery method	Trifunov et al. (2018)
Single‐cell mtDNA + ATAC	Mitochondrial genotyping combined with chromatin accessibility	Per‐cell heteroplasmy plus cell state; native lineage tracing	Allele dropout and uneven coverage; demanding informatics	Lareau et al. (2021), Ludwig et al. (2019)
Single‐cell mtDNA from scRNA‐seq	Enrichment of mtDNA variants from droplet scRNA‐seq	Clonal resolution within transcriptomic atlases	Limited to expressed and adequately covered positions	Miller et al. (2022)
Long‐read/iMiGseq	Full‐length single‐molecule mtDNA sequencing	Haplotype‐resolved heteroplasmy and linkage of variants	Lower throughput; higher input; PCR‐amplified (bias‐prone)	Bi et al. (2023)
mtDNA base editing with single‐cell tracking	DdCBE/TALED‐installed variants tracked per cell over time	Causal test of drift versus selection on defined variants	Engineering and off‐target constraints; experimental only	Guo et al. (2023), Kotrys et al. (2024)
Native long‐read (Cas9‐guided nanopore)	Cas9‐guided nanopore sequencing of native, unamplified mtDNA	Amplification‐free, full‐length, haplotype‐resolved sequencing; low bias	High input DNA; uneven coverage	Keraite et al. (2022), Vandiver et al. (2022)

Abbreviations: ATAC, assay for transposase‐accessible chromatin; DdCBE, DddA‐derived cytosine base editor; NGS, next‐generation sequencing; NUMT, nuclear mitochondrial DNA segment; scRNA‐seq, single‐cell RNA sequencing; TALED, transcription activator‐like effector‐linked deaminase.

## 7 Clinical and Translational Dimensions

### 7.1 From Mutation to Dysfunction: Linking the Clock to Aging Phenotypes

**FIGURE 4:**
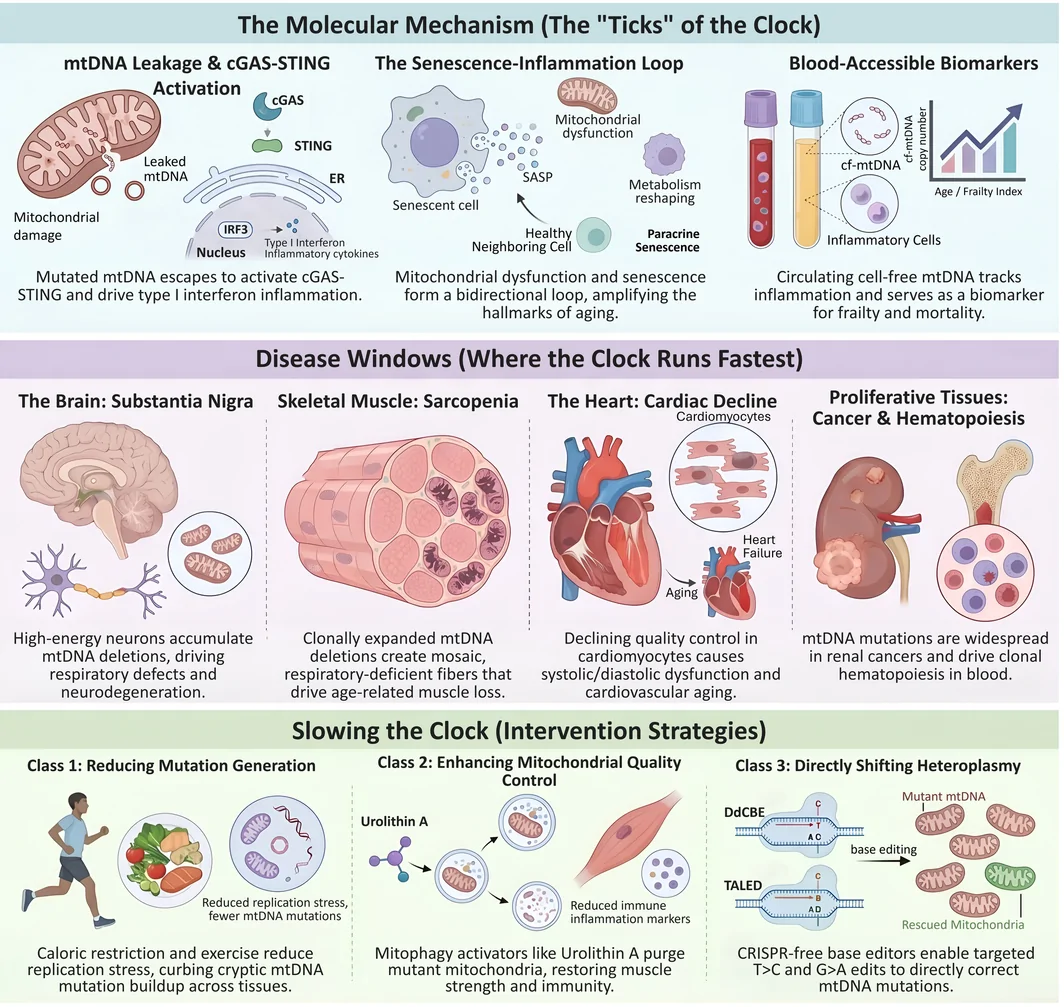
Clinical and translational dimensions of the mitochondrial clock. Mechanistically, leaked mtDNA activates cGAS–STING, feeds a senescence–inflammation loop, and yields blood‐accessible biomarkers; clinically, the clock runs fastest in brain, muscle, heart, and proliferative tissues. Interventions span three classes: Reducing mutation generation, enhancing mitochondrial quality control, and directly shifting heteroplasmy by base editing.

A clock is most useful if its ticks are mechanistically connected to the process it measures, and several pathways link clonally expanded mtDNA mutations to aging phenotypes (Figure 4). First, because of its bacterial ancestry, mtDNA is sensed as a damage‐associated molecular pattern when it escapes the organelle, activating the cGAS‐STING pathway and related sensors to drive Type I interferon responses and inflammation (Hu and Shu 2023). Mutational load and loss of mtDNA integrity promote this leakage, and the resulting signaling has been tied to the senescence‐associated secretory phenotype, positioning the mtDNA‐cGAS‐STING axis as a regulator of both inflammation and senescence in age‐related disease (Wen et al. 2026). Second, mitochondrial dysfunction and cellular senescence are deeply interconnected: dysfunctional mitochondria promote a senescent, secretory state, and senescence reshapes mitochondrial metabolism (Miwa et al. 2022). The accumulation of high‐heteroplasmy, respiratory‐deficient cells therefore feeds the senescent‐cell burden of aging, and mitochondrial dysfunction interacts bidirectionally with the other aging hallmarks, amplifying the consequences of any single defect (Li et al. 2024). Third, chronic low‐grade inflammation, or inflammaging, is a hallmark of aging and a driver of cardiovascular disease and frailty (Ferrucci and Fabbri 2018); circulating cell‐free mtDNA rises with cellular damage and correlates with inflammatory markers and adverse outcomes in older adults (Nidadavolu et al. 2023), and within‐individual declines in leukocyte mtDNA copy number interact with inflammation to predict mortality (Wu et al. 2026). These blood‐accessible mitochondrial measures connect the cellular clock to organismal outcomes.

### 7.2 Disease Windows: Where the Mitochondrial Clock Runs Fastest

If the mitochondrial clock advances at tissue‐specific rates, its consequences should surface first where energy demand is highest, cellular turnover is most constrained, or clonal selection is most intense. Several age‐related diseases can be read as windows in which the clock runs fast or its readout becomes clinically visible, and in each the underlying logic is the clonal mosaicism developed above rather than a uniform decline in mitochondrial mass. The clearest neuronal window is the substantia nigra, whose dopaminergic neurons accumulate clonally expanded mtDNA deletions to high levels during normal aging, with deletion burden directly implicated in the respiratory defect (Bender et al. 2006). More broadly, single‐cell analyses show that cryptic mtDNA mutations correlate with markers of neurodegeneration in aged neurons and can be slowed by caloric restriction (Green et al. 2025), and these observations sit within a wider literature linking central‐nervous‐system mitochondrial aging to Alzheimer's, Parkinson's and motor‐neuron disease (Steffan et al. 2025). The post‐mitotic, high‐energy character of neurons places them firmly in the hotspot‐biased arm of the biphasic clock (Wang, Li, et al. 2025), explaining why the brain is among the first organs in which clonal mtDNA damage becomes pathophysiologically informative.

Skeletal muscle provides the corresponding window in a renewable‐but‐aging tissue. Sarcopenia, the age‐related loss of muscle mass and strength, is tightly coupled to mitochondrial decline, and a defining histological feature of aged muscle is the mosaic of respiratory‐deficient, atrophic fibers driven by clonally expanded mtDNA deletions (Larsson et al. 2019). Because muscle loss is a principal driver of frailty, falls, and loss of independence, the muscle arm of the mitochondrial clock connects a molecular process directly to a major determinant of healthspan. Proliferative tissues expose a third window through cancer and clonal hematopoiesis. Pan‐cancer whole‐genome sequencing shows that mtDNA is pervasively mutated, that most variants are heteroplasmic, and that truncating mutations are enriched and positively selected in kidney, colorectal, and thyroid cancers, implying oncogenic consequences (Yuan et al. 2020); the proliferative arm of the clock therefore overlaps the very substrate from which tumors arise (Green et al. 2025; Wang, Li, et al. 2025). In blood, the same age‐accumulating heteroplasmy is mechanistically tied to clonal hematopoiesis, with germline modifiers near TERT and related loci shaping mutation burden (Gupta et al. 2026), and longitudinal single‐cell tracking directly captures the expansion of mutant hematopoietic clones that the clock model predicts (Lareau et al. 2019). The aging heart illustrates the post‐mitotic arm with particular clarity. Cardiomyocytes combine extreme, sustained energy demand with minimal turnover, precisely the conditions under which clonally expanded mtDNA mutations accumulate at recurrent hotspots and respiratory‐deficient cardiomyocytes appear as a mosaic in aged myocardium. Declining mitochondrial quality control in these cells contributes to diastolic and systolic dysfunction, and improving mitochondrial quality can attenuate age‐related cardiac decline in preclinical models and improve cardiovascular biomarkers in humans (Liu et al. 2025), tying the cardiac arm of the clock to a leading cause of death in older adults. Finally, the clock reads out as graded clinical risk at the level of the whole organism. Heteroplasmy burden is associated with increased all‐cause and cancer‐specific mortality (Hong et al. 2023), and recent work links accumulated heteroplasmic mutations to the severity of chronic kidney disease and to the risk of acute kidney injury, mechanistically through suppressed purine metabolism that is amenable to nuclear‐encoded metabolic rescue (Huang et al. 2025). These associations establish that the mitochondrial clock is not merely descriptive but is coupled to the hard outcomes that any clock of biological age is ultimately meant to predict.

### 7.3 Slowing the Clock: Prospects for Intervention

If clonal mosaicism behaves as a clock, the natural question is whether it can be decelerated. Candidate interventions fall into three broad classes: reducing the generation of new mutations, improving the clearance of mutation‐bearing mitochondria, and directly correcting or shifting heteroplasmy. Each engages a different point in the clock's mechanism, and each carries distinct evidence and limitations. The first class targets mutation generation through lifestyle and metabolic interventions. Caloric restriction slows the accumulation of cryptic mtDNA mutations across single cells in multiple tissues (Green et al. 2025), and exercise improves skeletal‐muscle mitochondrial capacity and energy metabolism in aging adults (Grevendonk et al. 2021). Importantly, because the dominant age‐accumulating signature reflects replication error rather than oxidative damage (Sanchez‐Contreras et al. 2023), strategies aimed narrowly at scavenging reactive oxygen species may be less effective than those that reduce replication stress or accelerate the turnover of defective genomes.

The second class enhances mitochondrial quality control to remove mutation‐bearing organelles, and here human evidence is now emerging. The mitophagy activator urolithin A improved muscle strength and endurance in randomized trials in middle‐aged and older adults (Singh et al. 2022), reduced markers of age‐related immune decline in a placebo‐controlled trial (Denk et al. 2025), and enhanced cardiac mitochondrial quality and cardiovascular biomarkers preclinically and in humans (Liu et al. 2025). By biasing the surviving mitochondrial pool toward functional genomes, such approaches could in principle slow the phenotypic readout of the clock even without lowering the underlying mutation rate, and they connect mechanistically to the senescence and inflammaging pathways through which mtDNA damage exerts its systemic effects (Ferrucci and Fabbri 2018; Hu and Shu 2023; Miwa et al. 2022).

The third and most direct class alters heteroplasmy itself. CRISPR‐free mitochondrial base editors based on a bacterial cytidine deaminase (DdCBE) enable programmable C‐to‐T edits in mtDNA (Mok et al. 2020), and TALE‐linked deaminases (TALED) extend programmable editing to A‐to‐G changes that DdCBEs cannot perform (Cho et al. 2022), while high‐fidelity engineered variants reduce off‐target activity (Lee et al. 2023). Together with nuclease‐based tools that selectively eliminate mutant genomes and shift heteroplasmy toward wild type, these technologies make the mtDNA population, in principle, a manipulable substrate. To date they have been applied chiefly to modeling and correcting inherited mitochondrial disease, are constrained by delivery and off‐target concerns, and remain proof‐of‐concept for somatic aging; whether they could ever be used to reset an aging somatic clock is an open and consequential question. A caveat applies across all three classes. Because the mitochondrial clock is tissue‐specific and partly under nuclear genetic control (Gupta et al. 2023), an intervention effective in one compartment may not generalize to others, and the appropriate endpoint, whether mutation rate, the per‐cell heteroplasmy distribution, or a downstream functional phenotype, must be matched to the mechanism being targeted. Defining these endpoints, and standardizing how the clock is read before and after intervention, is a prerequisite for testing whether mitochondrial aging can be therapeutically slowed.

## 8 The Mitochondrial Clock Among the Clocks of Aging

The dominant quantitative clocks of biological age are epigenetic, based on age‐correlated DNA methylation. The mitochondrial clock is distinct in origin and complementary in information: it reads accumulated somatic mutation in a physically separate, high‐copy genome with its own replication and selection rules. The two are coupled at the cellular level: iPSC‐derived cells engineered to high deleterious heteroplasmy show accelerated epigenetic age in their differentiated progeny, though this has so far been shown only in this pluripotent stem‐cell model rather than in somatic tissue in vivo (Vandiver et al. 2025), and emerging population data separately link mtDNA variants to epigenetic and biological aging measures even in young adulthood (Mareckova et al. 2025), suggesting that a combined mitochondrial‐plus‐epigenetic estimator might outperform either alone. Several features define the niche of the mitochondrial clock. It is intrinsically tissue‐specific and biphasic, so a single universal calibration is inappropriate and the choice of tissue is itself informative (Sanchez‐Contreras et al. 2023; Wang, Li, et al. 2025). It is partly under nuclear genetic control, making inter‐individual differences in clock rate heritable and potentially druggable (Gupta et al. 2023). It is causally linked to mortality and to organ‐specific disease rather than being purely descriptive (Hong et al. 2023; Huang et al. 2025). And it is readable in blood with maturing single‐cell and rare‐variant technologies, giving it translational potential (Lareau et al. 2021; Miller et al. 2022). Against these strengths stand real challenges: the cross‐sectional accumulation curve is steep only late in life unless rare‐variant methods are used; not all accumulated variants are functionally equivalent, so weighting schemes are required; bulk measurements blur the per‐cell distribution on which the clock depends; and the relative contributions of inherited baseline, de novo somatic mutation and clonal expansion must be disentangled before any reading is unambiguous (Green et al. 2025; Gupta et al. 2026).

Among existing clocks, those built on DNA methylation remain the best‐validated estimators of biological age and mortality, and the theory underpinning them is well developed (Horvath and Raj 2018). The mitochondrial clock is unlikely to replace them; its value lies in capturing an orthogonal axis of somatic damage in a genome that is uniquely traceable at single‐cell resolution and mechanistically tied to bioenergetic failure. Population data already link mtDNA variants to epigenetic and biological aging measures even in young adulthood (Mareckova et al. 2025), strengthening the case for integrated, multi‐omic clocks that read the nuclear and mitochondrial genomes together. Several controversies remain unresolved and temper any premature clinical claim. The relative weight of neutral drift versus active selection in shaping somatic heteroplasmy is still debated and appears to differ by tissue and proliferative status (Kotrys et al. 2024). The functional significance of the predominantly low average heteroplasmy seen in bulk tissue continues to be questioned, and is only partly reconciled by per‐cell and threshold considerations (Hinton Jr. et al. 2024). Whether the accumulation is best understood as a cause, a consequence, or a passenger marker of broader aging processes is not fully settled, given that much of the burden behaves as replication‐error passengers carried by clonal expansion (Sanchez‐Contreras et al. 2023). Acknowledging these open questions is essential, because the strength of the mitochondrial clock as a concept is that it renders them empirically tractable rather than rhetorical.

## 9 Conclusions and Future Directions

Evidence from single‐cell sequencing of thousands of genomes per cell, multi‐tissue duplex‐sequencing surveys, and biobank‐scale analyses converges on a coherent picture: somatic mtDNA mutations arise throughout life as cryptic, largely replication‐error events, expand clonally within individual cells under a combination of drift and selection, and accumulate in a tissue‐specific, biphasic manner that tracks chronological age and predicts mortality and organ‐specific disease. These are the defining properties of a molecular clock, and recognizing heteroplasmy as structured, interpretable information reframes a long‐running debate about whether mtDNA mutations “cause” aging into a more productive question about how the clock can be read, calibrated and slowed.

Several priorities follow. First, the biphasic clock should be formally parameterized across tissues, ideally through longitudinal single‐cell heteroplasmy measurement in accessible compartments such as blood, so that per‐cell mutational burden can be converted into a calibrated estimate of biological age. Second, the functional weighting of variants, integrating constraint scores, hotspot status, lesion class (point mutation versus deletion), and tissue context, needs standardization so that deleterious and neutral accumulation, as well as point‐mutation and deletion burden, are not conflated. Third, the mechanistic links from clonal mosaicism to inflammaging, senescence and organ failure should be exploited to test whether limiting mtDNA mutagenesis, enhancing mitochondrial quality control, restraining mtDNA‐driven inflammation, or nuclear‐encoded metabolic rescue can decelerate the clock. Finally, the heritable, nuclear‐encoded control of clock rate offers genetic entry points to the modifiers of mitochondrial aging. If these challenges are met, the mitochondrial clock will move from an explanatory framework to a practical, blood‐readable instrument for measuring how, and how fast, an individual is aging.

Viewed together, these strands recast mitochondrial aging from a vague decline into a measurable, mechanistically grounded process. The mitochondrial genome is, uniquely, a high‐copy somatic recorder that can be traced cell by cell, accumulates damage in a tissue‐specific and partly heritable manner, advances within individuals over time, and is causally tied to mortality and organ‐specific disease. Whether or not somatic mtDNA mutation is a primary driver of aging, clonal mosaicism of heteroplasmy is an unusually legible record of how that process unfolds, and the tools to read and potentially to perturb it now exist. Unlocking its promise will depend less on discovering new phenomena than on the unglamorous work of calibration, standardization, and longitudinal validation that turns a compelling biological signal into a dependable clock.

## Author Contributions

R.C., A.P.T., and C.‐J.L. conceived the overall concept of the study, while A.P.T. and B.W. contributed to the development of the original idea. R.C. and C.‐J.L. prepared the manuscript draft, and R.C., A.P.T., C.‐Y.P. and C.‐J.L. participated in the review and revision of the manuscript. All authors discussed the results and contributed to the final version of the manuscript.

## Funding

This research was funded by the National Science and Technology Council (114‐2628‐B075‐B‐001‐MY3) and Kaohsiung Veterans General Hospital (KSVGH‐114‐056, 148).

## Conflicts of Interest

The authors declare no conflicts of interest.

## Data Availability

No new data were generated or analyzed in support of this research.
